# Deep learning-based defects detection of certain aero-engine blades and vanes with DDSC-YOLOv5s

**DOI:** 10.1038/s41598-022-17340-7

**Published:** 2022-07-29

**Authors:** Xubo Li, Wenqing Wang, Lihua Sun, Bin Hu, Liang Zhu, Jincheng Zhang

**Affiliations:** 1 School of Automation, Xi’an University of Posts and Telecommunications, Xi’an 710121, China; 2 College of Astronautics, Nanjing University of Aeronautics and Astronautics, Nanjing, 211156 China

**Keywords:** Computer science, Aerospace engineering

## Abstract

When performed by a person, aero-engine borescope inspection is easily influenced by individual experience and human factors that can lead to incorrect maintenance decisions, potentially resulting in serious disasters, as well as low efficiency. To address the absolute requirements of flight safety and improve efficiency to decrease maintenance costs, it is imperative to realize the intelligent detection of common aero-engine defects. YOLOv5 enables real-time detection of aero-engine defects with a high degree of accuracy. However, the performance of YOLOv5 is not optimal when detecting the same defects with multiple shapes. In this work, we introduce a deformable convolutional network into the structure of YOLOv5s to optimize its performance, overcome the disadvantage of the poor geometric transformability of convolutional neural networks, and enhance the adaptability of feature maps with large differences in the shape features. We also use a depth-wise separable convolution to improve the efficiency of multichannel convolution in extracting feature information from each channel at the same spatial position while reducing the increased computational effort due to the introduction of deformable convolution networks and use k-means clustering to optimize the size of anchor boxes. In the test results, mAP50 reached 83.8%. The detection accuracy of YOLOv5s for common aero-engine defects was effectively improved with only a 7.9% increase in calculation volume. Compared with the metrics of the original YOLOv5s, mAP@50 was improved by 1.9%, and mAP@50:95 was improved by 1.2%. This study highlights the wide application potential of depth science methods in achieving intelligent detection of aero-engine defects. In addition, this study emphasizes the integration of DDSC-YOLOv5s into borescope platforms for scaled-up engine defect detection, which should also be enhanced in the future.

## Introduction

As the main power source of airplanes, normal operation of an aero-engine is the primary prerequisite for flight safety. Because an aero-engine works in an extreme environment with high temperature, high stress, and high speed and under the joint action of a working load and vibration load, severe environmental and operating conditions may result in increasing component defects, the engine is prone to failure. Meanwhile, foreign object damage may further harm the engine and the aircraft^1–3^. Due to the high cost of aero-engine maintenance^4^ and the strict requirements of workers and work sites for dismantling and assembling aero-engines, borescope inspection is widely used in condition-based maintenance. Borescope inspection is performed by professionals to detect the internal condition of the engine through borescope instruments. Borescope inspection of defects inside an aero-engine is usually performed manually via video, which not only requires the inspector’s expertise but also makes the result susceptible to subjective experience of the operator. Meanwhile, because of the complex and changeable background and the lack of light inside the engine, the inspector needs to manipulate the probe lens angle during the inspection to obtain good quality images. This slows the inspection process, and then tiny defects are easily missed by experienced inspectors, which causes intensive work with low efficiency^5,6^. Therefore, we couple deep learning with computer vision, apply an object detection method to aero-engine borescope inspection to detect defects, and use an improved YOLOv5s^7^ algorithm to locate and identify common aero-engine defects.

During the borescope process, the internal condition of the aero-engine is collected through an endoscope and output in the form of images without disassembling the engine^8^. At present, the relevant research on aero-engine defect detection mainly involves the implementation of trough images. Kim et al.^9^ used image-processing techniques to preprocess aero-engine blade borescope images and then used a CNN to classify them as normal or damaged. Image-processing techniques are used in the preprocessing step. They employ a scale invariant feature transform to extract the feature points in images and a K-dimensional tree and random sample consensus to match the feature points. The portion selected through the matching is a suspected damaged region. The CNN in the research consists of two convolution layers, and two fully connected layers are designed and trained to classify the result of the preprocessing. The method shows an average of more than 95% accuracy. Guo et al.^10^ utilized structural adaptive neural networks to realize the automatic recognition of tip curl, corrosion, crack and tear damage in engine borescope images through texture characteristics of the damages. In their research, the BP algorithm of a neural network was used to learn the internal connection and the weight parameters of the model and then obtain adaptive selection of network model parameters using a genetic algorithm. The recognition accuracy of the damage could be as high as 82%. Although damage classification and recognition were achieved^9,10^, failed to locate the damage. Shen et al.^11^ utilized FCN^12^ to identify and locate damages from borescope images. Semantic segmentation was selected in their framework for efficient thin crack and corrosion detection, though there was no model improvement. Classification accuracy was up to 90% in their experiments. He et al.^13^ proposed a network based on an improved cascade Mask R-CNN to identify the damage and its location while segmenting the area of damage on aero-engine blades. They used multiscale training to improve the network to obtain richer feature maps and to enhance the ability of the network to perceive damaged areas of aero-engine blades using a convolutional block attention module. The accuracy for gap and coating damage detection could reach 98.81%. Despite the high accuracy of the test, the disadvantage of^11,13^ was that the detection was too slow to detect in real-time. Zhang et al.^14^ applied the original YOLOv3 algorithm to the detection of aero-engine blade crack, crul, dent, nick and tear damage. Although the algorithm provided a good tradeoff between detection accuracy and detection speed reaching those of real-time detection, the accuracy was not high enough.

To address the deficiencies of the abovementioned research, we propose an improved YOLOv5 method named DDSC-YOLO for aero-engine defect detection. Considering several aspects of shape feature extraction, computational volume and detection performance, we embed DCN^15^ into different positions of the YOLOv5s network structure through multiple experimental comparisons to find the best position where deformable convolution is fully functional. The position offset in deformable convolution is implemented by a convolutional layer, and this convolutional operation increases the computational effort. We introduce DSC to reduce the computational effort caused by the offset operation of deformable convolution and extract more channel features. We also conduct several experiments to adjust the order of DCN and DSC embedding positions in the network structure to achieve improved optimal performance of YOLOv5. To obtain anchor boxes that better fit the engine defect objection, the variational operation of the genetic algorithm is used to improve anchor boxes clustered by K-means and is finally applied to engineering practice.

## Method description

**Figure 1:**
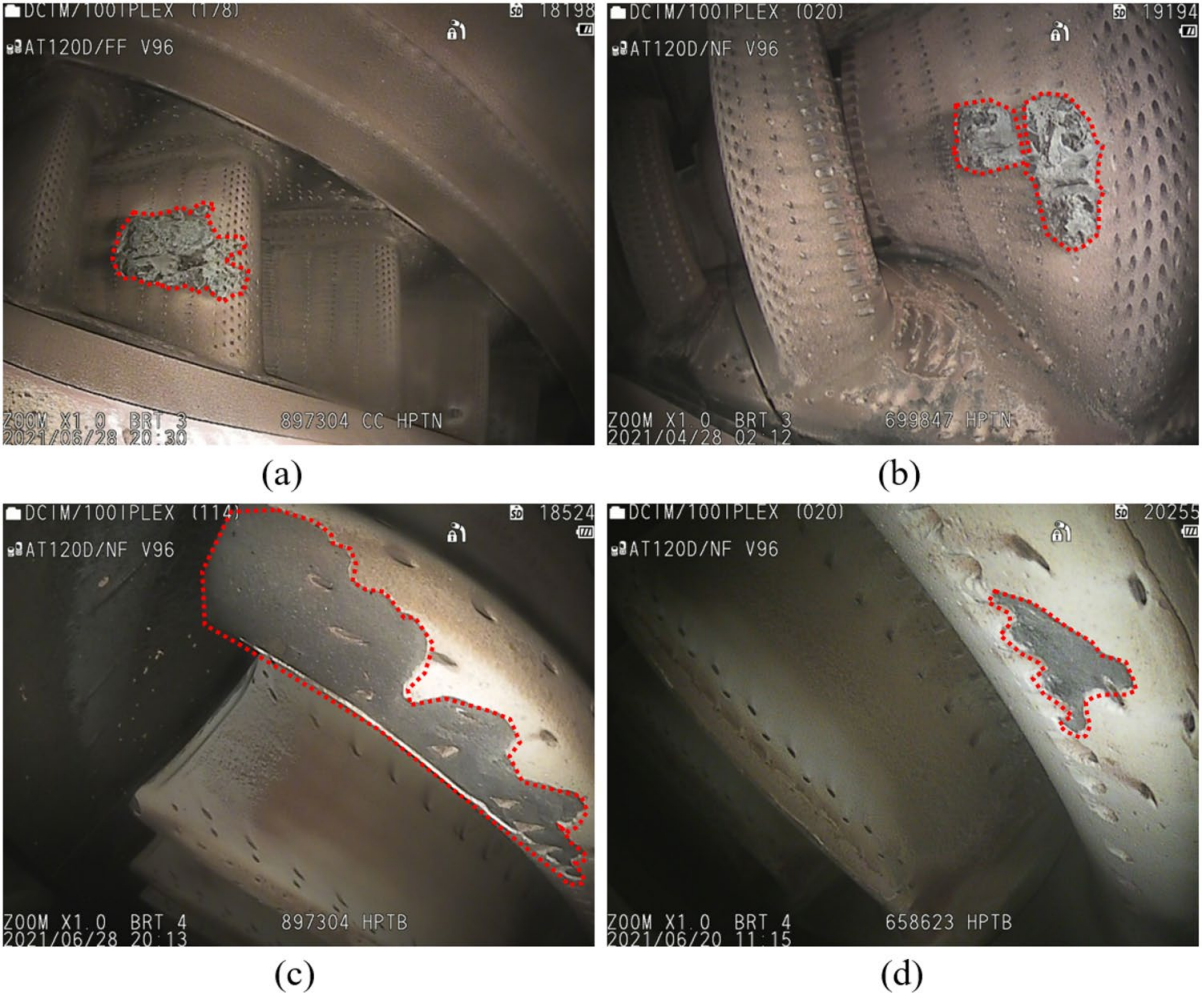
Same type of defect of different shapes. (**a**, **b**) are corrosion, (**c**, **d**) are the TBC missing.

Common objects of the same type, such as cars, trees and dogs, all have similar shapes. However, certain defects of the same type of aero-engine have different shapes, as shown in Fig. 1. Enhancing the extraction of defect shape features can make the defect features more comprehensive and reduce the classification error of the model due to the large difference in defect shapes. The innovation of this paper is to use deformable convolution to overcome the disadvantage of the poor geometric transformability of convolutional neural networks and enhance the adaptability of feature maps with large differences in shape features, including corrosion and missing TBCs.

We apply object detection to aero-engine maintenance and use YOLOv5s to achieve defect discrimination during borescope inspection. You only look once^16^ (YOLO) is a classical one-stage object detection algorithm that treats object detection as a regression algorithm that takes the whole picture as input to realize object detection globally. Compared to two-stage object detection algorithms, the background error of YOLO is lower. YOLO series algorithms^16–19^ are all based on YOLO. YOLOv5 was proposed by ultralytics. The official code has 4 versions: Yolov5s, Yolov5m, Yolov5l, and Yolov5x. YOLOv5s is the selected version for the experiment in our method, and we improve its structure with the DCN and DSC as follows.

### Deformable convolution

**Figure 2:**
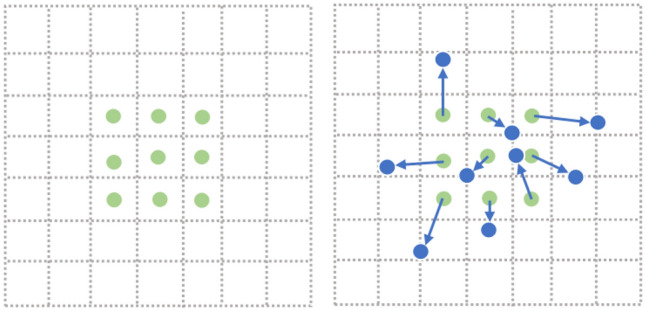
Illustration of the sampling locations in 3 × 3 standard and deformable convolutions. The left shows the regular sampling of 9 points for conventional convolution, and the right shows deformed sampling locations (dark blue points) with augmented offsets (light blue arrows) in deformable convolution.

An inherent drawback of conventional convolution is that only fixed positions of the input feature map can be sampled. Deformable convolution introduces the ability to learn spatial geometric deformation by adding a positional offset to the position of each sampling point in the kernel, which enables kernel sampling at random around the current position and weakens the regular grid point sampling limit of conventional convolution in a CNN. Figure 2 is the comparison between the sampling points of conventional convolution and deformable convolution^15^.

The conventional convolution operation samples the input feature map with a regular grid *R*, and then extracts features by weighting the sampled values with a weight *w*. In the deformable convolution operation, an offset {${\varvec{p}}_{{\varvec{n}}} |\varvec{n} = 1, \, 2, \ldots ,N$} is added to the regular grid *R*, where ${\varvec{N}} = \left| {\varvec{R}} \right|$. For each deformable convolution kernel, there are sampling points ${\varvec{p}}_{0}$, and ${\varvec{p}}_{{\varvec{n}}}$ is used to represent the offset of each sampling center point. Thus, the sampling is performed at irregular and offset positions ${\varvec{p}}_{{\varvec{n}}}$ + $\Delta {\varvec{p}}_{{\varvec{n}}}$. The output feature y can be expressed as follows:

1 $${\varvec{y}}\left( {{\varvec{p}}_{0} } \right) = \sum \limits_{{{\varvec{pn}} \in {\varvec{R}}}} {\varvec{\omega}}\left( {{\varvec{p}}_{{\varvec{n}}} } \right) \cdot {\varvec{\chi}}\left( {{\varvec{p}}_{0} + {\varvec{p}}_{{\varvec{n}}} + \Delta {\varvec{p}}_{{\varvec{n}}} } \right)$$

**Figure 3:**
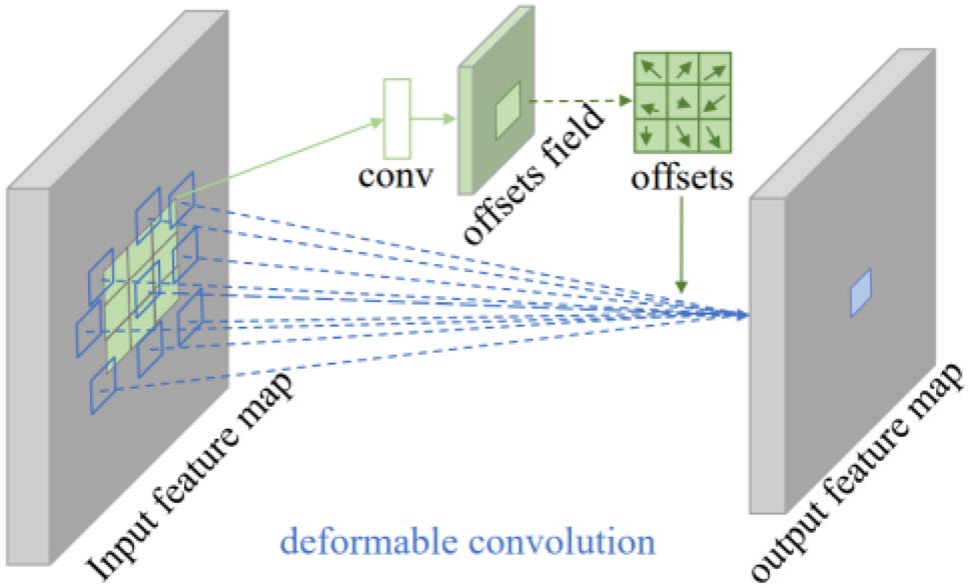
3×3 deformable convolution process.

As shown in Fig. 3^15^, the offset in a 3 × 3 deformable convolutional network is obtained through a common convolutional layer. The offset field with the number of output channels 2N is the same size as the feature map with an input channel of N. N represents the size of the convolution kernel, whose value is k × k. 2N corresponds to two-dimensional offsets of N in the x and y directions. The conventional convolution that generates offset and deformable convolution of the output feature are trained at the same time. To learn the offsets, the gradient is propagated backward by a bilinear interpolation operation.

### Depth-wise separable convolution

Conventional convolution uses the same kernel of all input channels for feature extraction, and the nondifferentiated treatment of input channels may prevent some key features from being extracted effectively. Depth-wise separable convolution adopts different kernels for various input channels, which reduces the redundant expression of kernels and effectively reduces the number of parameters, thus reducing the computational effort. Depth-wise separable convolution can be implemented by two processes: depth-wise convolution and pointwise convolution.

**Figure 4:**
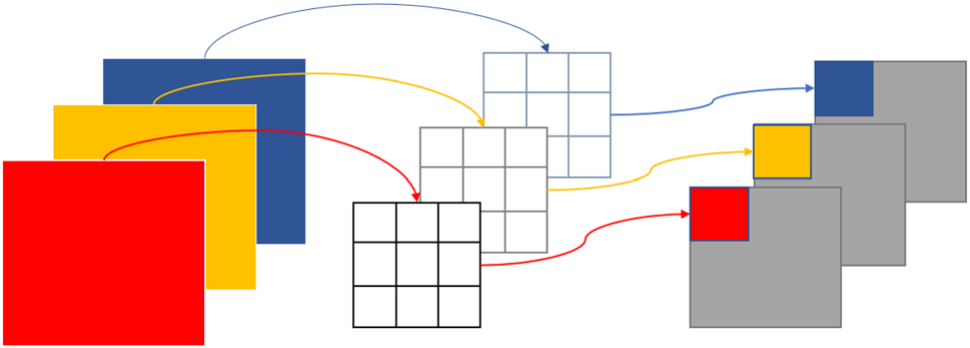
Depth-wise convolution process.

As shown in Fig. 4, during depth-wise convolution, one kernel extracts features for one channel, and feature maps of input channels correspond to kernels one-to-one. Therefore, the number of output channels in the depth-wise convolution process is the same as the number of input channels. The number of feature maps obtained after depth-wise convolution is the same as the number of input channels, so feature maps cannot be extended. The convolution operation is performed independently on each channel of the input layer, and the feature information of the different channels at the same spatial location cannot be used effectively. Therefore, pointwise convolution is needed to combine these feature maps to generate a new feature map.

**Figure 5:**
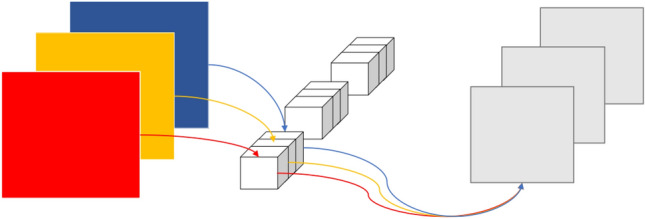
Pointwise convolution process.

The pointwise convolution operation can be regarded as a conventional convolution operation with a kernel size of 1 × 1. The feature map processed by depth-wise convolution is subjected to pointwise convolution to ensure that the feature map processed by depth-wise convolution is weighted and combined in the depth direction to generate a new feature map. The number of output features is the same as the number of kernels. The pointwise convolution process can be seen in Fig. 5.

## Improved YOLOv5s network

Most object detection backbone networks use deep learning networks to extract features. As the most widely used deep learning network, convolutional neural networks (CNNs) can effectively extract image features, but CNN-based object detection is not the best approach. Deformable convolution enhances the CNN's modeling ability for deformed objects^15^ and extracts image features better. Adding a DCN to the YOLOv5s network structure overcomes the shortcomings of the poor geometric transformability of CNNs and enhances the ability to adapt feature maps with large differences in shape features. Conventional convolution extracts features in one convolution, but depth-wise separable convolution extracts features by two convolutional layers, one for extracting features and the other for combining features from different channels. This factorization can effectively reduce the computational effort and the size of the trained model. This paper improves YOLOv5s to propose an aero-engine defect detection method. Based on the original YOLOv5s, the conventional convolution module of the network is replaced by the DConv module for the DCN introduced into YOLOv5 and DSConv to introduce DSC.

**Figure 6:**
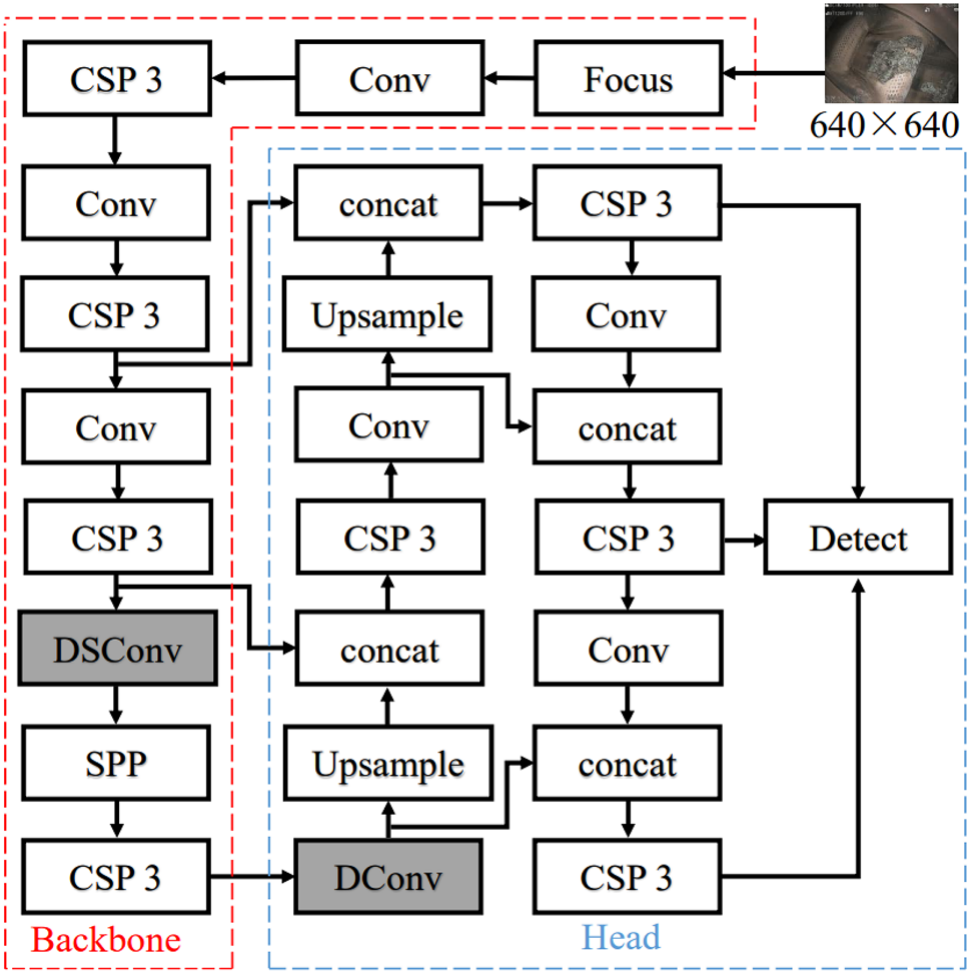
Improved YOLOv5s network structure.

We performed a series of experiments in the previous period. When the DConv module is placed in front of the structure, because the feature map contains too many features, the increased offset of the DCN for extracting deformed features increases the computation to the extent that the GPU cannot operate. When the DConv module is placed further back in the structure, the feature map loses most of the deformation features, so the DCN cannot extract enough deformation features to be useless. Ultimately, we found that placing the DConv module at the intersection of the YOLOv5 backbone and head resulted in the most substantial improvement in the performance of the original network. Similarly, after determining the location of the DConv module, we conducted several experiments by placing the DSC module at different locations of the network and found that the performance of YOLOv5s improved more when placed in front of the DConv module than when placed at back. Eventually, the improved YOLOv5s structure with the best performance was determined, as shown in Fig. 6.

**Figure 7:**
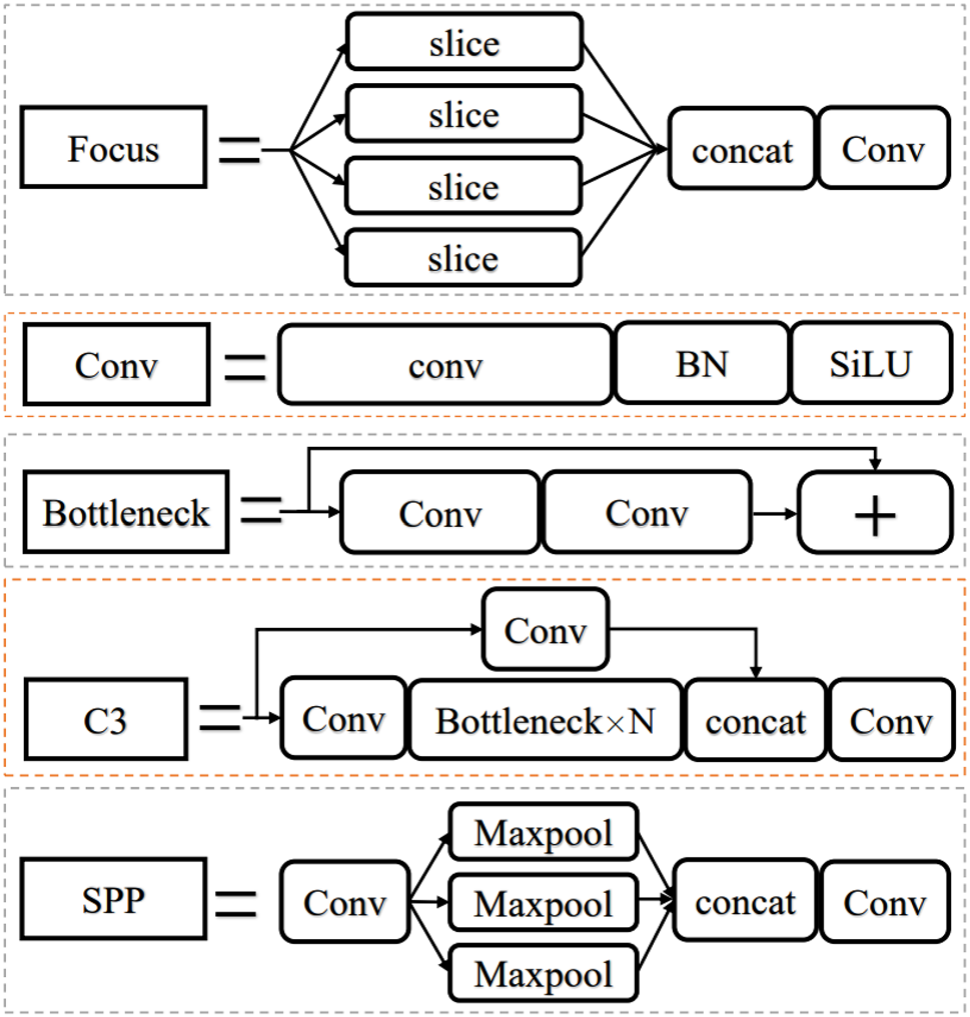
The composition of each module of YOLOv5s.

The backbone of the network consists of 1 focus module, 3 conv modules, 4 CSP3 modules and 1 SPP module. The principle of each module can be seen in Fig. 7. The focus module slices the input picture to convert it shape from 640 × 640 × 3 into 4 feature maps of 320 × 320 × 3, which achieves downsampling without losing feature information from the picture and extracts it for rich information while speeding up training. The Conv module is a CNN operation with batch normalization (BN) and is activated by the SiLU activation function. The CSP 3 module is a CSP bottleneck with 3 convolution operations; it is a cross-stage local network that can reduce the number of parameters and ensure the inference speed. The SPP module is based on the idea of a spatial pyramid network^20^, which realizes the simultaneous extraction of local features and global features, which makes it beneficial for large differences in target size in the image to be detected. The head of the network implements feature fusion at different scales by convolution, upsampling and concatenation. In the detection part, anchor boxes are applied on the feature map, and a final output vector with class probabilities and enclosing frames is generated.

### DConv module

**Figure 8:**
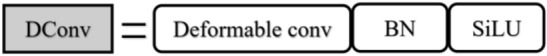
DConv module structure.

After extracting effective features from the input image by the backbone, deformable convolution is used to further extract the features with position offset, and the resulting features are normalized by BN and then activated by the SiLU activation function. The above process is performed by the DConv module, which is shown in Fig. 8. The DConv module gives YOLOv5 deformable convolution capabilities to enhance the adaptability of the network to features with large differences in shape features, and it can extract features more effectively. Using deformable convolution with a kernel size of 1 × 1 to calculate the offset of sampling points tends to lead to sampling instability. However, the larger the kernel is, the more calculations there are. Therefore, we use a 3 × 3 deformable convolution kernel for feature extraction with a stride of 1 and the same number of input and output channels, both of which are 256.

### DSConv module

**Figure 9:**
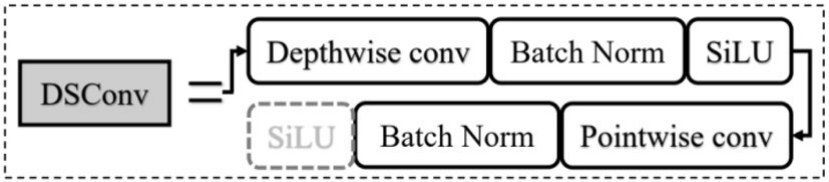
DSConv module structure.

The function of the DSConv module is to implement a depth-wise separable convolution consisting of depth-wise convolution and pointwise convolution. Depth-wise convolution can be realized by group convolution with the same number of input and output channels. The input channels and the output channels of conventional convolution are divided into 256 groups for convolution computing with a kernel size of 3 × 3 and a stride of 2. The resulting features are batch normalized, and then activated with the SiLU function for the pointwise convolution operation. The input channel is 256, the output channels are 512, and the stride is 1. After batch normalization, selective activation is performed by the SiLU function. The process is illustrated in Fig. 9.

## Cluster anchor boxes

### The common aero-engine defect dataset

Modern aircraft engines mostly use gas turbines with a complex load and structure; harsh working environments cause various types of defects. In the daily operation of certain airline fleets, common defects include cracks, dents, corrosion, missing material, and a missing TBC. The explanations for the defects are shown in Table 1.

**Table 1:** Defect type and its explanations.

Type	Explanations
Crack	A slender slit, caused by uneven distribution of thermal stress or foreign object impact, mostly in the hot zone (the tip of the high-pressure turbine rotor blade). Cracks may extend to cause material loss
Dent	A small, smooth indention with rounded edges, corners and bottom caused by mechanical impact of a dull object. Often, dents can be found at or close to the blade edges
Corrosion	Shape extremely irregular burn damage, mostly occurs on the inner and outer walls in high temperatures of the back burning area of the combustion chamber
Missing material	By the high speed of the larger volume of foreign objects or internal falling hard object impact caused by the blade part of the material loss, often occurs in the multistage blade
TBC missing	Very irregular shape of the thermal barrier coating missing, mostly occurs in the edge of the vane, if not timely treatment will lead to vane ablation

A total of 850 pictures of 5 types of defects from CFM aero-engine borescope images of airlines are collected and divided into a training set, validation set, and test set according to the ratio of 8:1:1. Table 2 shows the defect type and sample quantity of the original dataset. After data augmentation of the training set through methods, such as color transformation, random color, flipping, rotation angle, size reduction, and noise addition; 13,600 images were finally obtained. Defect objects are labeled by LabelImg software^21^.

**Table 2:**
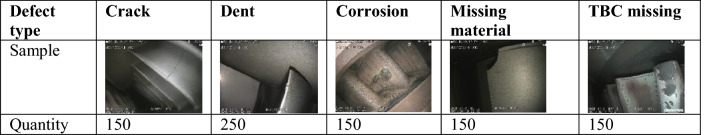
Defect type and sample quantity of the original dataset.

### Cluster anchor boxes

YOLOv5 automatically calculates the best possible recall (bpr) of the default anchor boxes and all object boxes in the dataset. If it is less than 0.98, it will recluster anchor boxes according to the object box of the dataset; otherwise, it will use the default anchor boxes. The bpr of the default anchor boxes of the dataset is 0.99, so the network uses the default anchor boxes. Due to large differences in the shape and size of defect objects in the aero-engine defect dataset and in the COCO dataset^22^, clustering is used to obtain the anchor boxes of aero-engine defects for better and faster aero-engine defect object matching. Then, the mutation operation of the genetic algorithm is used to optimize the anchor boxes obtained by clustering to improve their fitness.

**Figure 10:**
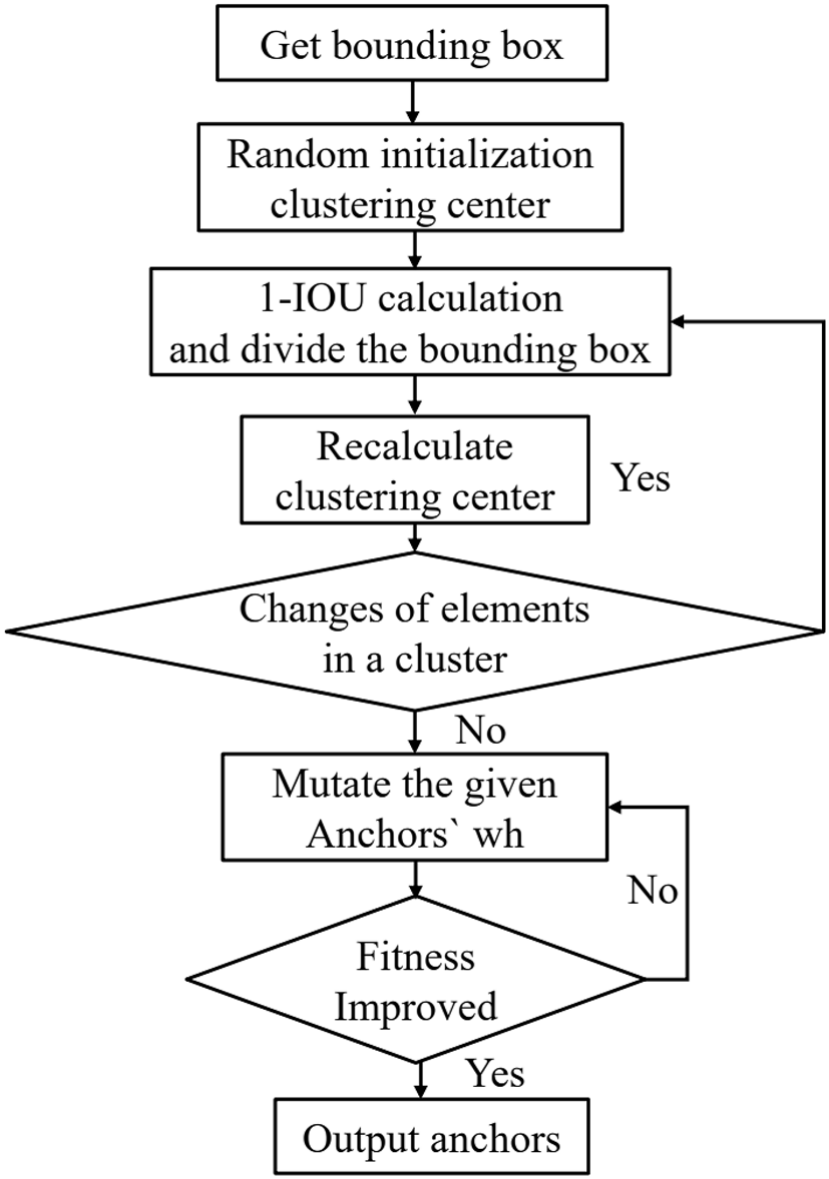
Clustering anchor boxes process.

The clustering process for the anchor boxes is illustrated in Fig. 10. First, we read and randomize the initial positions of all bounding boxes and select 9 boxes as cluster centers. Then, we calculate the distance (1-IOU) of each object bounding box from each cluster center, where IOU is the intersection ratio of bounding boxes and anchors of each object. Next, the cluster center with the closest distance to each object bounding box is calculated, and each object bounding box is assigned to the cluster closest to it. Finally, the cluster centers are recalculated based on the object bounding boxes in each cluster. The above process is repeated until the elements in each cluster no longer change, and then the obtained anchor boxes are mutated to select the anchor boxes with the highest fitness.

**Figure 11:**
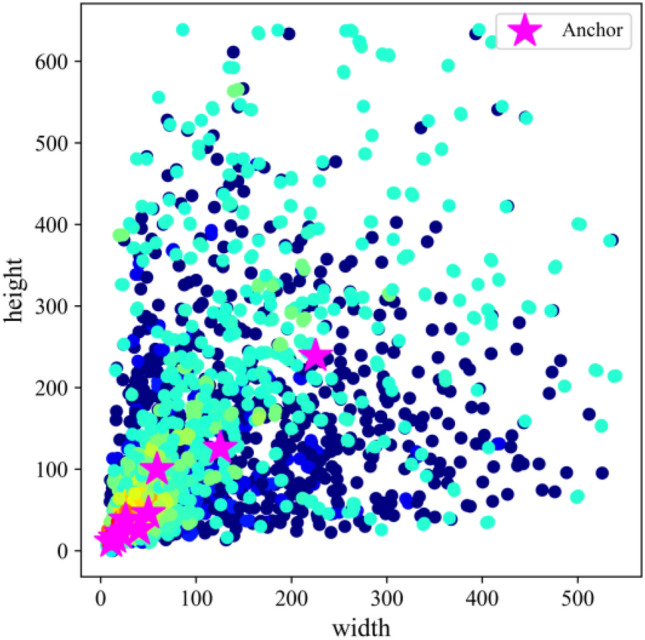
The size of the object boxes and the obtained anchor boxes.

The width (w) and height (h) of the bounding box in each picture of the training set are read, and the maximum values of w and h are scaled equally to 640. Then, k-means clustering is used to obtain 9 anchors, and the genetic algorithm randomly mutates a total of 18 gene segments of anchors w and h. The fitness of the anchors is obtained after the mutation is evaluated. If the fitness is better after mutation, the result will be assigned to the anchors, and if the effect becomes worse, we skip it. After a mutation operation of times 1000, the anchors obtained by the final mutation are sorted and output according to the size of the area. We select the anchor boxes with the highest fitness during several clustering operations: [10,12] [16,16] [20,23] [38,26] [26,39] [49, 46] [65,100] [141,124] [219,267], determined as the final anchor boxes after random mutation: [11,11] [15,16] [21,22] [26,38] [40,26] [50,46] [59,100] [125,126] [225,239]. Figure 11 shows the sizes of the object boxes and the obtained anchor boxes.

## Experiment and result analysis

### Evaluation index

The experimental results are evaluated from 8 perspectives: mAP@50, mAP@50:95, Precision, Recall, average detection processing time, parameter amount, computation volume and model size.

Precision (P) is defined as the proportion of true positive (TP) samples among all predicted positive samples, which include false-positive (FP) samples. Recall (R) is defined as the percentage of all TP samples among the predicted positive samples, which include false negative (FN) samples. TP means prediction is true and actually true. FP means prediction is true but actually false. FN means prediction is false but actually true. The precision and recall are calculated using formulas^5^ (2) and (3) as follows:

2 $$\varvec{ P} = \frac{{{\varvec{TP}}}}{{{\varvec{TP}} + {\varvec{FP}}}}$$

3 $$\varvec{ R} = \frac{{{\varvec{TP}}}}{{{\varvec{TP}} + {\varvec{FN}}}}$$

AP stands for average precision, which integrates the performance of both Precision-Recall and Recall, expressed as the area of the region surrounded by Precision-Recall curves and coordinate axis. As a measure of detection accuracy in object detection, mean average precision is abbreviated as mAP and is calculated by summing the average accuracy of all categories and dividing it by the number of categories. IoU refers to the intersection ratio of the ground truth bounding box to the prediction bounding box. mAP@0.5 is the mAP when the IoU threshold is set to 0.5. mAP@0.5:0.95 indicates the average mAP at different IoU thresholds (from 0.5 to 0.95 in 0.05 units). The AP and mAP calculations comply with formulas^23^ (4) and (5) as follows:

4 $$\varvec{ AP} = \int \limits_{0}^{1} {\varvec{PdR}}$$

5 $${\varvec{mAP}} = \frac{{ \sum \nolimits_{{{\varvec{i}} = 1}}^{{\varvec{N}}} {\varvec{AP}}_{{\varvec{i}}} }}{{\varvec{N}}}$$

where R denotes the expression of the precision-recall curve. N is the number of object categories, and the value of N is 5 in this experiment.

### Model training environment

The experiments are conducted using the PyTorch1.7.1 deep learning framework implemented in Python. The platform is a 64-bit Windows 10 operating system with cuda10.1 and cudnn10.1. The processor is an Intel(R) Xeon(R) CPU E5-2620 v4 @ 2.10 GHz, NVIDIA GeForce GTX1080Ti graphics card, 11G video memory. The training process uses the Adam optimizer and sets the initial learning rate to 0.01, weight attenuation to 0.0001, momentum to 0.9, and batch size to 32. The training process uses the pretrained model YOLOv5s trained from the COCO dataset to initialize the weights, training for 150 epochs.

### Model training analysis

To verify the effect of the introduction of deformable convolution, depth-wise separable convolution, and cluster anchor boxes on the performance of YOLOv5s and the role of each module, experiments are conducted on a self-built common aero-engine defect dataset. The following model is used for training:

1. YOLOv5s is compared with other improved models to highlight the performance optimization of each improved model. As a reference group, the performance of this model is used to analyze the effect of introduction for each module on the original model.
2. DCNv1-YOLOv5s is the only deformable convolutional network introduced into the head of YOLOv5, while just the DConv module is embedded into the YOLOv5s network structure, and the position is shown in Fig. 6. The original network backbone is not optimized.
3. DCNv2-YOLOv5s are the only deformable convnets v2^24^ that are introduced into the head of YOLOv5s, and only the DConv module is embedded into the YOLOv5s network structure, as shown in Fig. 6. The original network backbone is not optimized.
4. DSC-YOLOv5s is the only depth-wise separable convolution that is introduced into the backbone of YOLOv5s, while only the DSConv module is embedded into the YOLOv5s network structure, and the position is shown in Fig. 6. It is only optimized for the backbone network of the original network, and the head is not optimized.
5. DDSCv1-YOLOv5s introduces deformable convolutional networks and depth-wise separable convolution into YOLOv5s. Both the DConv module and the DSConv module are embedded in the YOLOv5 network structure at the same time. The SiLU activation function is used to activate at the end of the DSC module. The embedding position is shown in Fig. 6. The backbone network of the original network is optimized by DSC, and the head is optimized by DCN at the same time.
6. DDSCv2-YOLOv5s introduces deformable convnets v2(DCNv2) and depth-wise separable convolution into YOLOv5s. Both the DConv module and the DSC module are embedded in the YOLOv5 network structure at the same time, but the SiLU function is not used for activation at the end of the DSC module. The position of the embed is shown in Fig. 6. The backbone network is optimized by DSC, and the head is optimized by DCNv2.
7. DDSCv1-Anchor-YOLOv5s introduces deformable convolutional networks and depth-wise separable convolution into YOLOv5s, and both the DConv module and DSC module are embedded in the YOLOv5s network structure at the same time. The SiLU function is used at the end of the DSC module for activation. The embedding position is shown in Fig. 6, using DSC to optimize the original network backbone and DCN to optimize the head at the same time. The anchor boxes obtained by K-means clustering are also used.
8. DDSCv2-Anchor-YOLOv5s introduces deformable convnets v2 and depth-wise separable convolution into YOLOv5s, and both the DCN module and DSC module are embedded in the YOLOv5s network structure at the same time. However, the SiLU function is not used at the end of the DSC module for activation. The embedding position is shown in Fig. 6, using DSC to optimize the original network backbone and DCNv2 to optimize the head at the same time. The anchor boxes obtained by K-means are used simultaneously.

**Figure 12:**
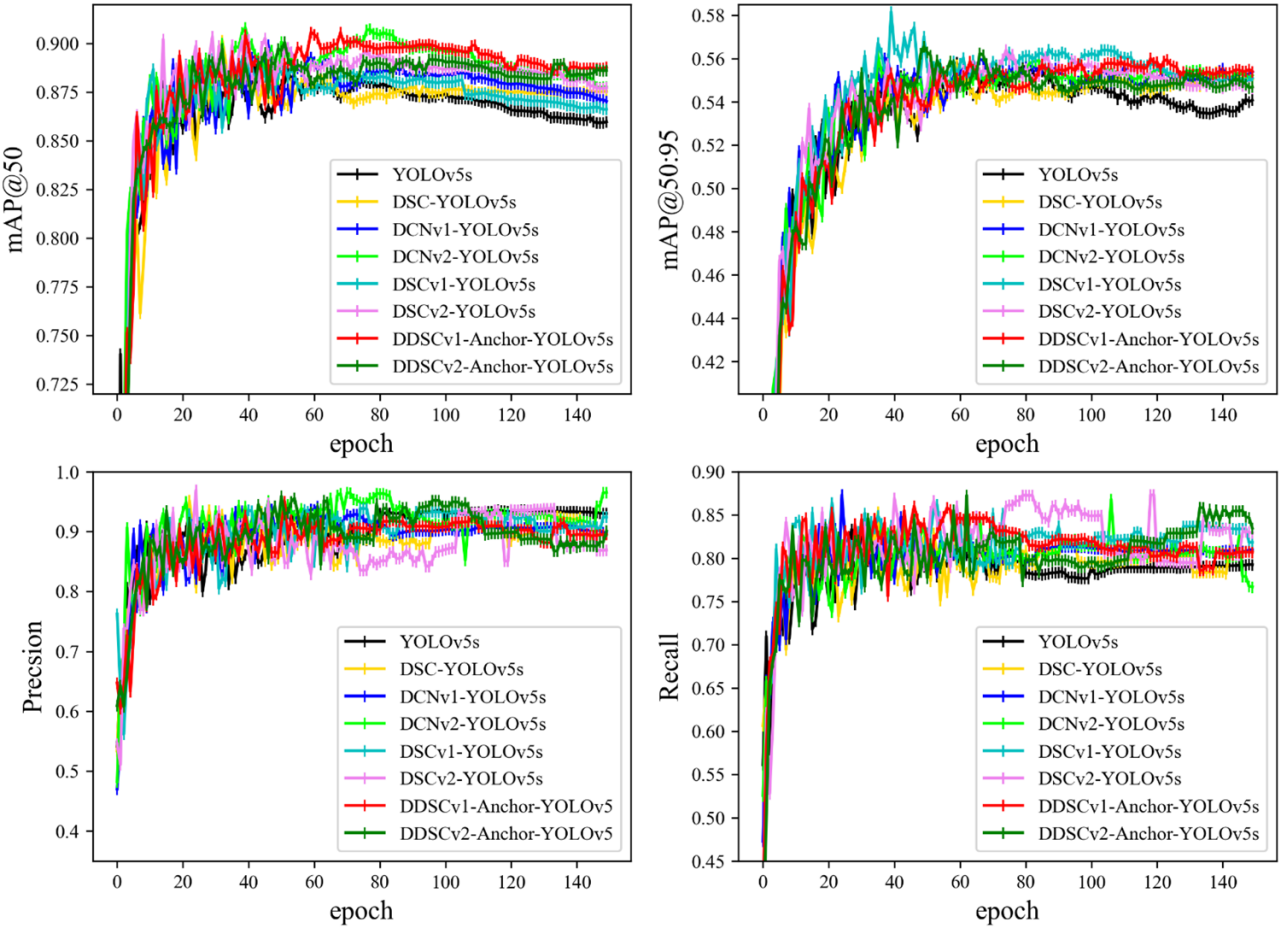
The parameter change curve of each model’s training process, except that some models’ precision is slightly lower than that of the original network. The mAP and the recall of the improved models are higher than those of the original network.

The mAP@50, mAP@50:95, precision and recall curves of the models in the training process are shown in Fig. 12, which indicates that during the training process, except for some models, the precision is slightly lower than that of the original network. The mAP and the recall of the improved models are higher than those of the original network. When comparing the models to each other, it was found that the optimization of YOLOv5s using deformable convolution, just depth-wise separable convolution or both can improve the detection accuracy of aero-engine defects, which proves the superior feature extractabilities of DCN and DSC when compared with conventional convolution. Among those models, the DCNv2 feature extractability is the best.

When deformable convolution and depth-wise separable convolution are used together, the feature extractability is substantial. On the one hand, it is because each convolution has stronger feature extractability compared with conventional convolution. On the other hand, the method of this paper fully considers the sequence of the two when used together. Placing DSC in the backbone can help the backbone network of YOLOv5s extract more channel features. Deformable convolution at the head of YOLOv5s configures position offsets for extracted multichannel features, enabling the network to extract more shape features.

In the literature^17^, YOLOv2 uses clustering to generate anchor boxes for the first time, but that reduces the mAP. However, the method in this paper uses cluster anchor boxes to improve recall and considerably increase the mAP, which proves the superiority of this method.

### Analysis of test results

The results of the test with each model obtained from training on the test set are shown in Table 3. The mAP from the test results is better than that of the training on the validation set during training. This is determined by the characteristics of the aero-engine defect dataset, which is the aero-engine defect training set characteristics of the decision. The location and shape contours of most defect objects in the aero-engine dataset are random and have large differences, which leads to overfitting of the training more easily. Except for DDSCv2-Anchor-YOLOv5, the remaining models are basically consistent with the result of the validation set in the training process. Considering that DCNv2 has the weights of position offsets, it is easy to cause more overfitting of the effective feature extraction at the same time.

**Table 3:** Test results of each model.

Model	DCNv1	DCNv2	DSC	Anchor	P (%)	R (%)	mAP@.5 (%)	mAP@.5:95 (%)
1					92.4	74.7	81.9	50.1
2	√				87.1	79.2	82.3	48.9
3		√			85.3	76.8	81.9	47.5
4			√		87.5	74.0	81.4	48.4
5	√		√		93.8	74.7	82.5	50.1
6		√	√		90.6	75.3	83.4	51.3
7	√		√	√	91.4	77.6	83.8	51.3
8		√	√	√	93.3	76.2	81.9	47.5

**Figure 13:**
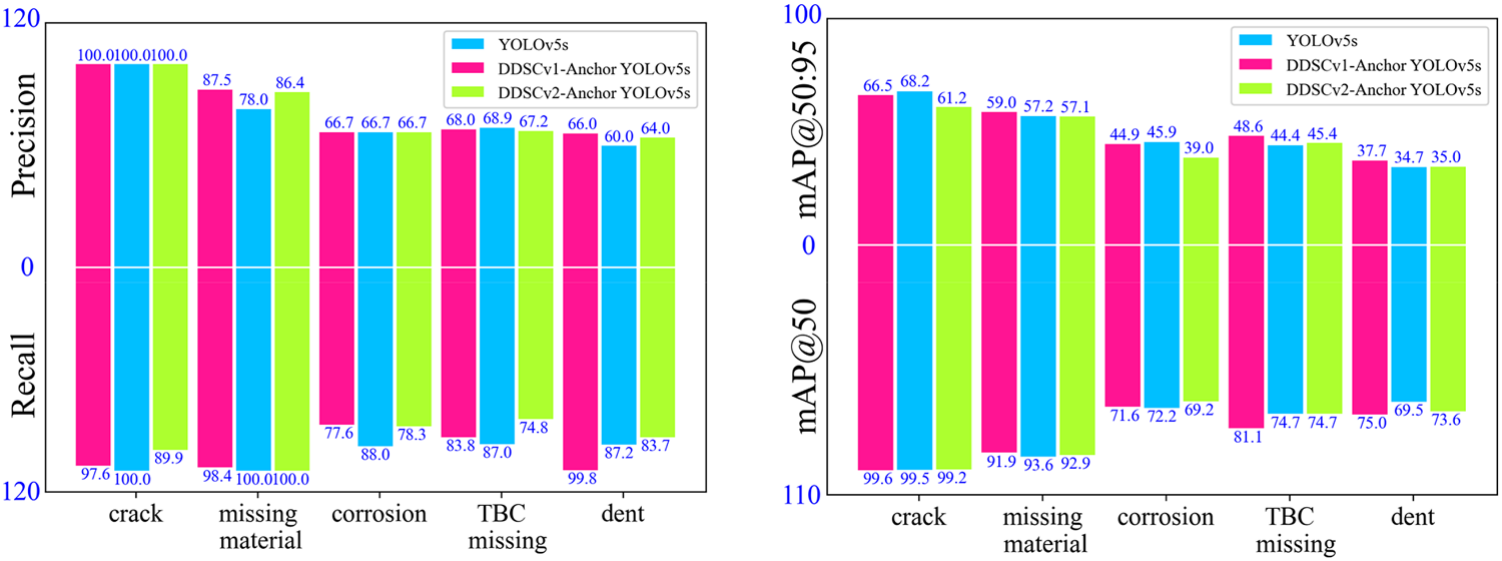
Precision, recall, mAP@50 and mAP@50:95 of different defects. The mAP improvement of the original YOLOv5s by the method in this paper is mainly reflected in the missing TBC and dent. Precision, mAP@50 and mAP@50:95 and recall of the dent have been improved considerably.

The method in this paper is based on DCN and DSC to improve YOLOv5s for common aero-engine defect detection with DDSCv1- Anchor-YOLOv5 and DDSCv2- Anchor-YOLOv5s as the final models. The bar chart of precision, recall, mAP@50 and mAP@50:95 is shown in Fig. 13. It can be seen that the mAP improvement of the original YOLOv5s by the method in this paper is mainly reflected in the missing TBC and dent. The most notable improvement in the mAP for the missing material is up to 9.5%. Precision, mAP@50 and mAP@50:95 and recall of the dent have been improved widely. The defect of the dent is characterized by a very small damage area, and it can be said that the method in this paper enhances the YOLOv5s’s detectability of small objects.

### Model performance

Table 4 shows the number of parameters and computation volume of each model, the weight size obtained by training and the average detection time of each image. Depth-wise separable convolution considerably reduces the number of parameters, calculation amount and weight size of YOLOv5s, but the average detection time increases. The introduction of deformable convolution increases the number of parameters, computation volume, weight and average detection time. The simultaneous use of deformable convolution and depth-wise separable convolution achieves a relative balance between computational effort and detection accuracy. Although the detection speed is reduced, it still does not affect the realization of real-time detection.

**Table 4:** Performance comparison of each model.

Model	Parameter	computation volume (GFLOPS)	Weights (MB)	Average detection time (ms)
YOLOv5	7,074,330	16.4	13.7	17.0
DCNv1-YOLOv5s	9,582,638	18.6	18.5	20.5
DCNv2-YOLOv5s	9,624,117	18.6	15.3	27.8
DSC- YOLOv5s	6,029,082	15.6	11.7	18.5
DDSCv1- YOLOv5s	8,537,388	17.7	16.5	23.3
DDSCv2- YOLOv5s	7,928,117	16.9	15.3	29.3
DDSCv1- Anchor-YOLOv5s	8,537,388	17.7	16.5	23.5
DDSCv2- Anchor-YOLOv5s	7,928,117	16.9	15.3	28.8

**Figure 14:**
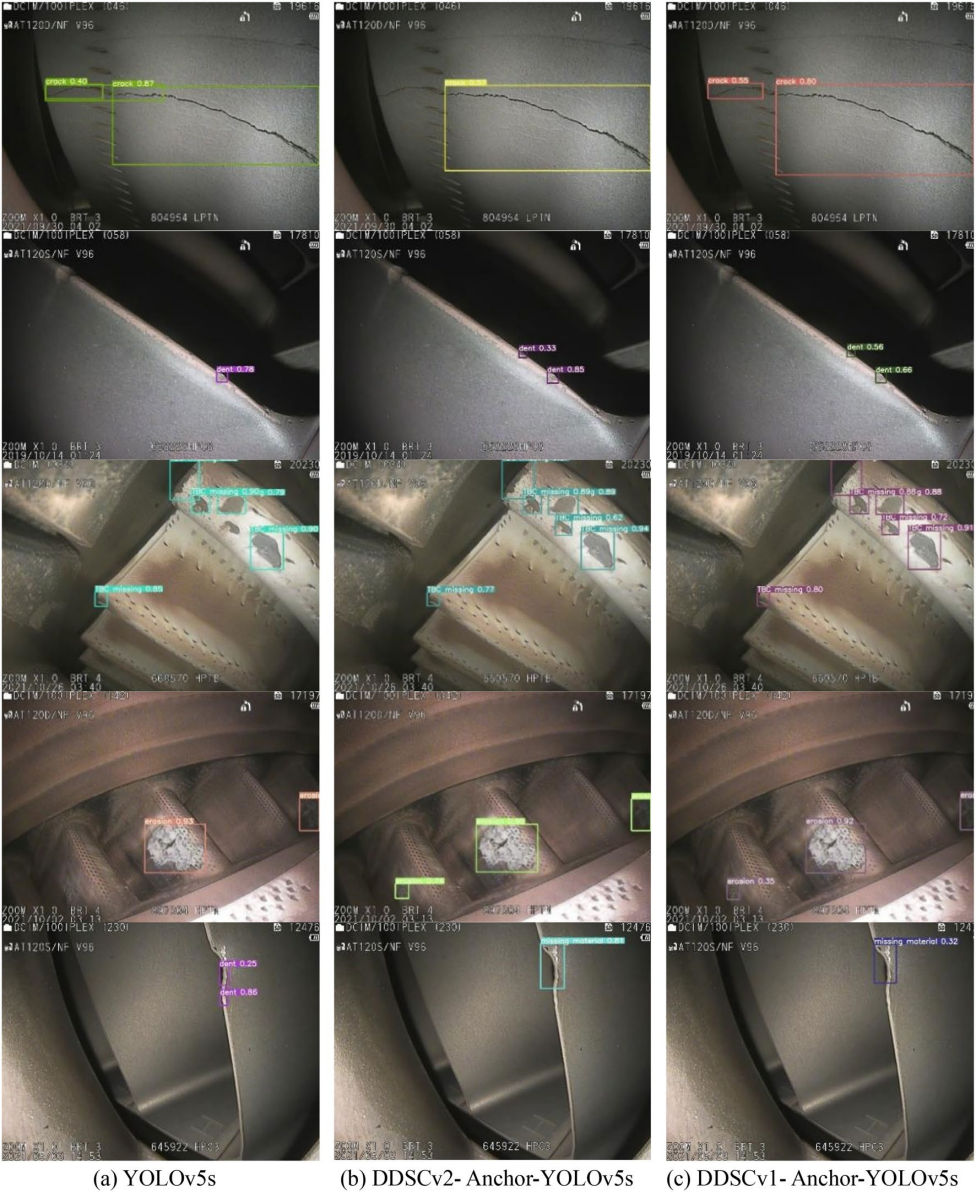
The performance of each model. The picture in the first row is the detection effect of the same crack that runs through the heat spreading hole of the blade. YOLOv5s detects one crack as three objects, DDSCv1-YOLOv5s does not detect a part of the crack on the upper side of the heat spreading hole, and DDSCv2-YOLOv5s has the most accurate detection effect. The second row of images shows the detection effect of the dents. It can be seen that both the YOLOv5s and the improved YOLOv5s are not very good at detecting small objects, but the performance of the improved model is still better than the original model. The third and fourth rows of images show the effect of the detection of missing TBCs and corrosion compared to the improved YOLOv5s and original YOLOv5s, which misses some objects. The fifth row of YOLOv5s misidentifies the missing material as the dent, and the bounding box is also inaccurate, while the improved YOLOv5s shows good performance.

Figure 14 shows the detection effect of the YOLOv5s and the improved YOLOv5s on common aero-engine defects. It can be seen that the improved model is more accurate in bounding boxes, reduces the missed detection of objects, and strengthens the detectability of small objects.

## Conclusion

As a result of the shortcomings of the skilled personnel requirements and low efficiency of aero-engine borescope inspection, we propose an improved YOLOv5 algorithm based on DCN and DSC to apply deep learning to aero-engine defect detection. The mAP@50 of YOLOv5s can be improved by 1.9% and mAP@50:59 by 1.2% on the test set of the self-built dataset, and the accuracy can basically meet the level required for engineering applications. Although the increase in computation leads to a slower detection speed, real-time detection is needed. Extracting more channel features using depth-wise separable convolution and learning the spatial geometric deformation of the feature map using deformable convolution can improve the detectability of YOLOv5s for small objects. This study can be a reference for improving other network structures by the cooperative use of the two and the sequential arrangement of each other.

Although the method improves the performance of the YOLOv5s to realize real-time detection of aero-engine defects, this research uses a self-built small dataset and only considers 5 types of defects of aero-engines, which need to be improved before they can be applied to engineering practice. Defect size is an important basis for determining the degree of aero-engine damage. Subsequent research will use deep learning to evaluate the damage level based on real-time detection of aero-engine defects.
